# Use of Wax and Resin Patterns in Global Fixed Prosthetic Rehabilitation

**DOI:** 10.1002/ccr3.70215

**Published:** 2025-02-11

**Authors:** Etienne Lefrançois, Ludovic Aubault, Salomé Provost

**Affiliations:** ^1^ Department of Prosthodontics University of Rennes, Pôle Odontologie, CHU Rennes, CNRS, ISCR (Rennes Institute of Chemical Sciences), UMR 6226 Rennes France; ^2^ Pôle Odontologie CHU Rennes Rennes France; ^3^ Department of Prosthodontics University of Rennes, Pôle Odontologie, CHU Rennes Rennes France

**Keywords:** computer‐aided design, computer‐aided manufacturing, dental porcelain, dental prosthesis, tooth preparation

## Abstract

In clinical situations of complete rehabilitation with fixed prostheses, mastering each parameter of all restorations (shape, marginal adaptation, proximal and occlusal contacts) proves to be a challenge. The large number of restorations involves many potential adjustments that are time‐consuming and lead to final ceramic properties degradation. This clinical report highlights the benefits of using castable patterns in complete fixed prosthetic rehabilitation. Minimal preparations of the teeth were carried out using the conventional mock‐up technique. The impressions were then digitized to produce wax and resin patterns (Castable Wax Resin; Formlabs) with a stereolithography 3D printer (Form 2; Formlabs). During oral try‐in of patterns, adjustments can be carried out to correct any defects of shape, marginal adaptation, proximal, and occlusal contacts. The definitive restorations were directly pressed from the patterns into lithium disilicate glass‐reinforced ceramic (IPS e.max Press; Ivoclar Vivadent) using the lost‐wax casting process. Finally, the restorations were placed permanently in the mouth with complete patient satisfaction after 4 years of follow‐up. This procedure is intended to reduce ceramic adjustments on definitive restorations. It is especially relevant in complete fixed rehabilitation and in situations where the thickness of ceramic restorations does not allow for try‐in before bonding.

## Graphical abstract

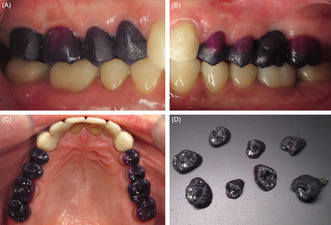

**Summary**

- The wax and resin patterns technique allows for convenient adjustments of patterns during oral try‐in, seamlessly incorporated into the final restoration fabrication, thus minimizing the need for ceramic adjustments.
- This preserves ceramic integrity and saves time, especially in complete fixed prosthetic rehabilitation, by preventing major defects that would require remanufacturing.

## 1 Introduction

Extended fixed prosthetic rehabilitation presents a challenge for the clinician due to the difficulty of achieving proper fit across multiple restorations. Over the last few decades, computer‐aided design and manufacturing (CAD/CAM) technology has transformed dental practice, offering more efficient procedures for prosthodontists and dental technicians. The accuracy of fixed CAD/CAM restorations can vary depending on factors such as impression techniques (conventional or digital), materials, manufacturing processes (such as milled or heat‐pressed ceramics), and operator experience [1, 2]. Despite this, CAD/CAM technologies generally provide clinically acceptable fixed restorations [2, 3, 4], often outperforming traditional methods [1, 2]. Digital tools, such as the virtual patient [5, 6, 7], guide treatment planning and the creation of final restorations. A diagnostic wax‐up determines optimal rehabilitation in the laboratory, and the mock‐up, clinically transposed in the mouth, serves as both an esthetic and functional template, helping to preserve dental tissue during preparation and acting as provisional restorations [8, 9, 10]. Depending on the manufacturing process, the final restorations may closely match the corrected and validated mock‐ups.

The evolution of 3D printing techniques and computer numerical control (CNC) milling offers numerous possibilities for manufacturing prosthetic restorations. Today, a wide range of printable and millable waxes and resins are commercially available for various applications, such as splints, interim restorations, or templates for press and casting techniques. On the one hand, there is a large variety of wax or resin discs available for milling castable patterns used in the production of fixed restorations. These materials can be composed entirely of wax or a mixture of wax and acrylic resin in varying proportions, with polymethyl methacrylate being the most common resin. A broad selection of material colors is available, ranging from basic shades (e.g., purple, red, blue, green, gray) to those that closely match dental tissue (e.g., VITA shades). On the other hand, 3D‐printable castable resins have been specifically developed for producing lost‐wax and heat‐pressed dental restorations. These resins are composed of either pure acrylic resin or a mixture of wax and resin in varying proportions. The color options are more limited (e.g., purple, black, blue, yellow, red), and to the best of our knowledge, no 3D printable castable material (clearly indicated by the manufacturer) in VITA shades is available on the market.

The choice of color and mechanical properties depends on the specific product, and these factors play a key role in their use, such as during clinical try‐in. Wax patterns have long been used in the mouth to check the fit of large metal frameworks [11] or as esthetic and functional testers in full mouth rehabilitations [12, 13]. These patterns can be used to either press or scan‐copy‐mill the final restorations [13]. For instance, milled mock‐ups have shown greater accuracy compared to conventional molded mock‐ups [14]. Conversely, printed mock‐ups have been reported to fit more accurately with the virtual design than milled mock‐ups [15, 16]. However, no detailed technique for the use of these patterns as clinical try‐ins and templates has been described so far.

In clinical situations requiring extensive fixed restorations, ceramic restorations are traditionally tried in the mouth, and any adjustments are made directly on the ceramic. Corrections by addition are almost impossible, and corrections by subtraction are associated with a risk of reducing the properties of the ceramic [17, 18, 19, 20, 21, 22]. The purpose of this article is to suggest a method for using CAD/CAM wax and resin patterns as a try‐in and a template for pressed ceramic restorations in a complete mouth rehabilitation, in order to reduce ceramic adjustments on final restorations.

## 2 Case History

**FIGURE 1:**
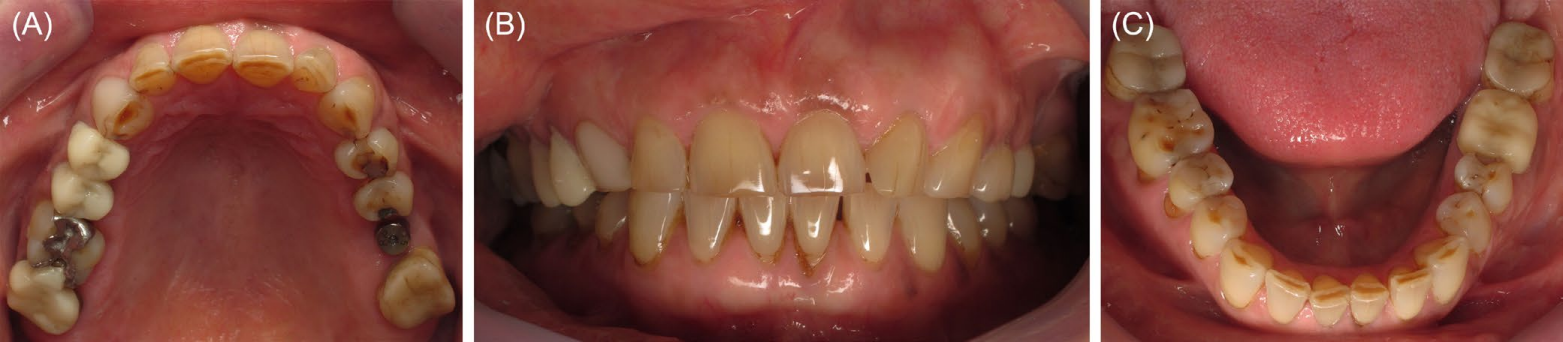
Initial intra‐oral situation of the 55‐year‐old woman, (A) maxillary occlusal view, (B) front view, and (C) mandibular occlusal view.

A 55‐year‐old woman presented with the chief complaint of missing upper left first molar #26 and the need for esthetic rehabilitation of the worn dentition. Clinical examination found a good oral hygiene with no dental plaque, no caries, a defective amalgam restoration on the upper right first molar #16, many previous aged direct restorations and fixed crowns, general attrition, abrasion, and erosion, and a recently placed implant at the edentulous area of tooth #26 (Figure 1). After discussion with the patient, the accepted treatment plan included prosthetic adhesive restorations of all maxillary and mandibular teeth (crowns, overlays, and veneers) and an implant‐supported crown for the missing tooth. The protocol is illustrated by focusing on the posterior maxillary restoration step integrated into the complete mouth treatment plan.

## 3 Methods

### 3.1 Complete Mouth Treatment Plan

The wax‐up was traditionally designed by the dental technician on a preliminary cast assembled on an articulator with a face bow. Modifying the occlusal vertical dimension (OVD) was guided by tissue preservation and the space required to restore optimal occlusal anatomy. In this clinical situation, the incisal pin of the articulator was increased to 3 mm to recover adequate height of OVD. In the posterior region, crowns were provided for teeth that previously had crowns or significant deterioration (#17, #15, #14, and #36), and overlays (with varying degrees of coverage) for the other teeth. In the anterior region, veneers are planned for the maxillary (full coverage of the entire tooth for canines) and mandibular anterior block from canine to contralateral canine. The chronological sequence of the treatment plan began with the mandibular rehabilitation, followed by the posterior maxillary rehabilitation, and finally the maxillary anterior rehabilitation.

**FIGURE 2:**
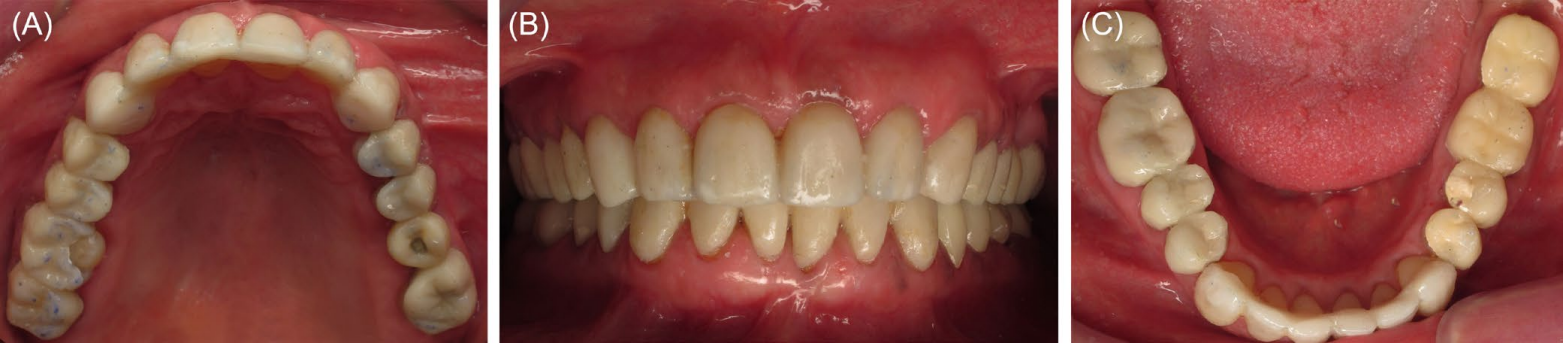
Chairside cemented mock‐up, (A) maxillary occlusal view, (B) front view, and (C) mandibular occlusal view.

Previously, silicone keys had been modeled on casts for iso‐molding (Aquasil Hard Putty; Dentsply Sirona). The project was tested in the mouth of the patient by transferring the wax‐up into a bisacryl resin (Dentocrown; Itena) mock‐up to evaluate esthetic and functional parameters in an initial approach. A new mock‐up was made from the keys in methacrylate resin (Unifast; GC). It was adjusted and then cemented with temporary cement (Temp Bond NE; Kerr). A temporary abutment (Temporary abutment engaging Conical Connection RP; Nobel Biocare) was used for the interim implant‐supported crown and was manually torqued. This complete mock‐up was kept in the mouth (Figure 2).

After 2 weeks of functional and aesthetic validation of the project by the patient, the mandibular rehabilitation was carried out first. The same protocol was used for the maxillary posterior rehabilitation and is detailed below.

### 3.2 Preparation of Posterior Maxillary Teeth

Posterior maxillary teeth were prepared through the mock‐up to preserve residual dental tissues. For ceramic adhesive restorations, the teeth were reduced to the minimum thickness recommended for monolithic pressed ceramic restorations in order to retain as much enamel as possible. Controlled penetration through the mock‐up was 1.5 mm on the occlusal surfaces and 0.5–1 mm on the other surfaces. If any part of the mock‐up remained after controlled penetration, the preparation was stopped once the enamel layer was reached. Resin (Duralay, Reliance) was inserted between the maxillary preparations and the restored mandibular teeth to register the maxillomandibular relationship (MMR). To maintain the validated OVD, the MMR was recorded on one side before removing the other posterior mock‐up side following the same protocol.

Complete maxillary impression was taken with polyvinylsiloxane silicone (Aquasil; Dentsply Sirona). New provisional restorations were made (Unifast; GC) for maxillary molars and premolars, adjusted to the preparations, and were cemented (Temp Bond NE; Kerr). An impression of the antagonist arch was taken with alginate (Hydrogum 5; Zhermack). All the elements were sent to the laboratory for the production of the patterns.

### 3.3 Patterns Laboratory Procedure

**FIGURE 3:**
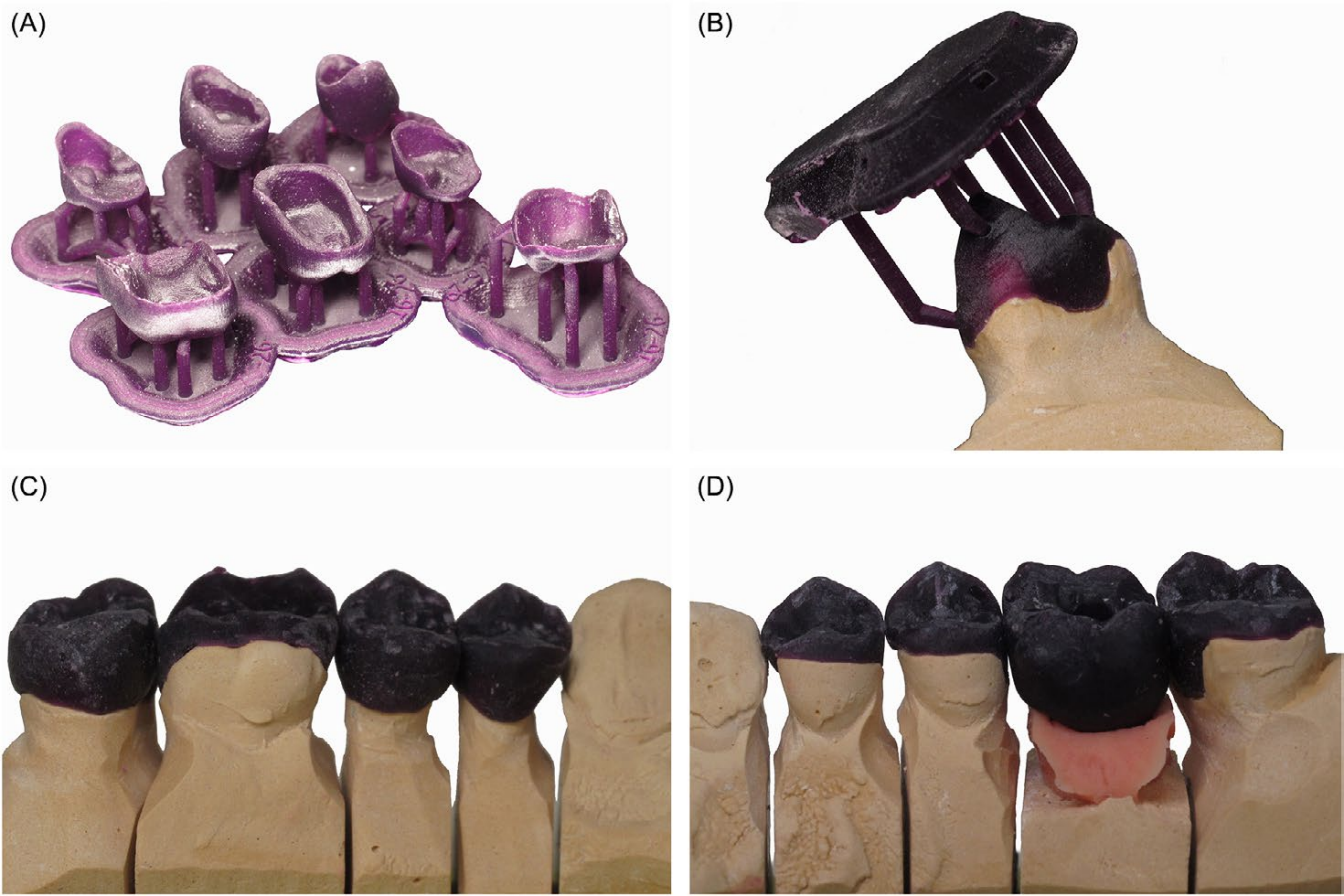
(A) Castable wax and resin mix printed patterns design for future restorations, directly after printing. (B) Fit of patterns on the dies with printing supports, on maxillary (C) right and (D) left molars and premolars.

Impressions were cast in plaster, cross‐mounted on an articulator (checked with the resin occlusal index) and then digitized with a laboratory scanner. Urethane dimethacrylate‐based castable wax and resin mix patterns (Castable Wax Resin; Formlabs) were digitally designed using a dental software and fabricated with a stereolithography 3D printer (Form 2; Formlabs) in accordance with the wax‐up. Then they were washed in isopropyl alcohol for 5 min. They were rinsed in a second bath to remove any residual uncured resin and finally light‐cured. They were adjusted on the plaster cast, and the print supports were removed (Figure 3). The resin pattern of the implant‐supported crown was set on a definitive implant abutment (Universal Base Conical Connection RP; Nobel Biocare) without bonding. Occlusal contacts were checked on the articulator.

### 3.4 Wax and Resin Patterns Try‐In

**FIGURE 4:**
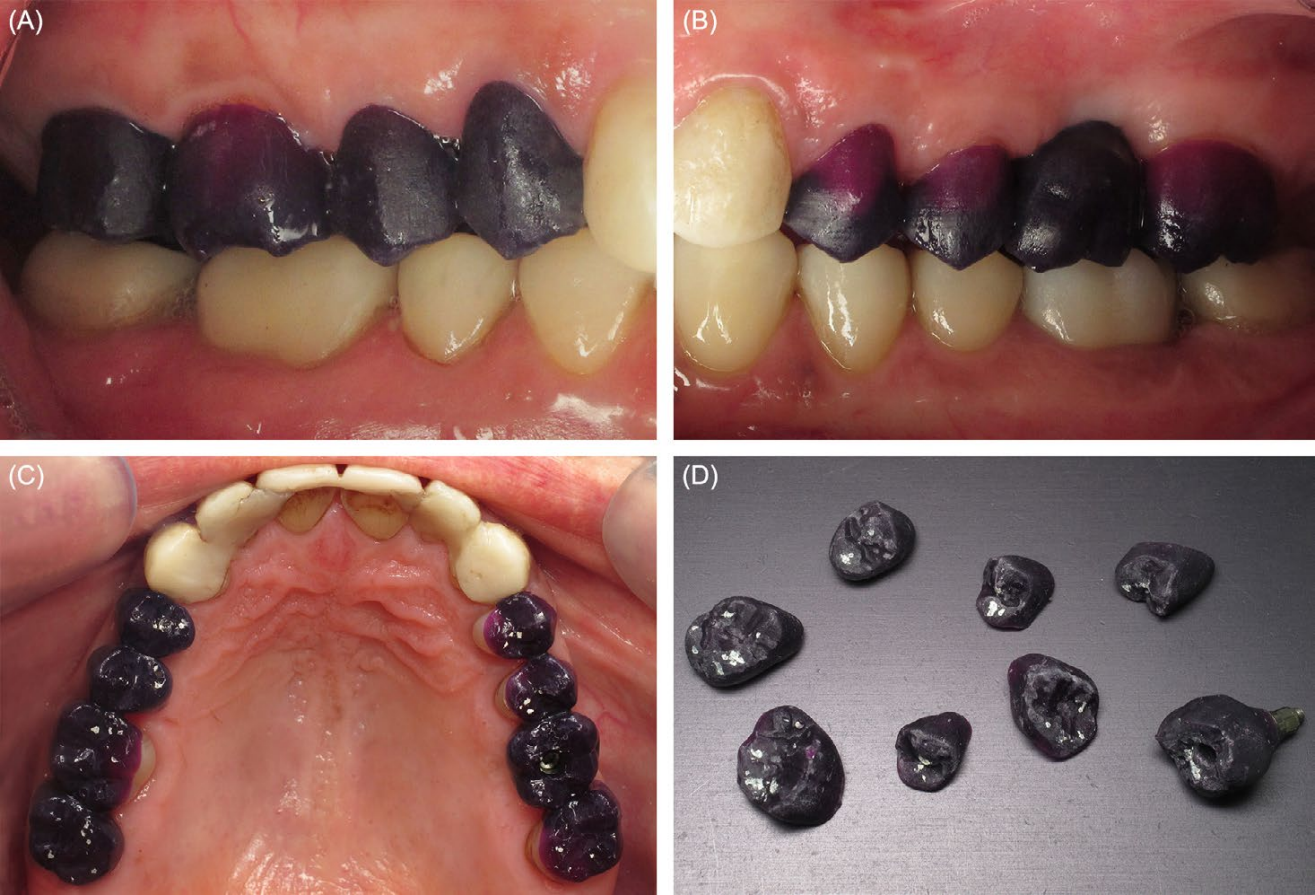
Patterns try‐in on maxillary (A) right and (B) left molars and premolars. (C) Occlusal contacts marked with 8 μm white articulating paper after occlusal adjustments. (D) Patterns once occlusal contacts have been marked and adjusted.

After removing the provisional restorations, the patterns were tried and adjusted in the clinical situation. Anatomy, cervical adjustment, proximal contacts, static, and dynamic occlusal contacts were checked (Figure 4). 8 μm white articulating paper (Articulating Film Ultra‐Thin 8 μ White; Baush) was used to detect precise occlusal contacts. In this situation, only a few occlusal adjustments were made by subtraction to optimize occlusal contacts. There was no modification of the shape or cervical limits of the patterns. Occlusal reduction can be carried out with a fine diamond bur, and contacts can be intensified by the addition of castable resin (Duralay; Reliance). Restorations are then disinfected and sent to the laboratory for manufacturing. The interim resin restorations were cemented again in the maxillary posterior region.

### 3.5 Manufacturing of Ceramic Restorations

**FIGURE 5:**
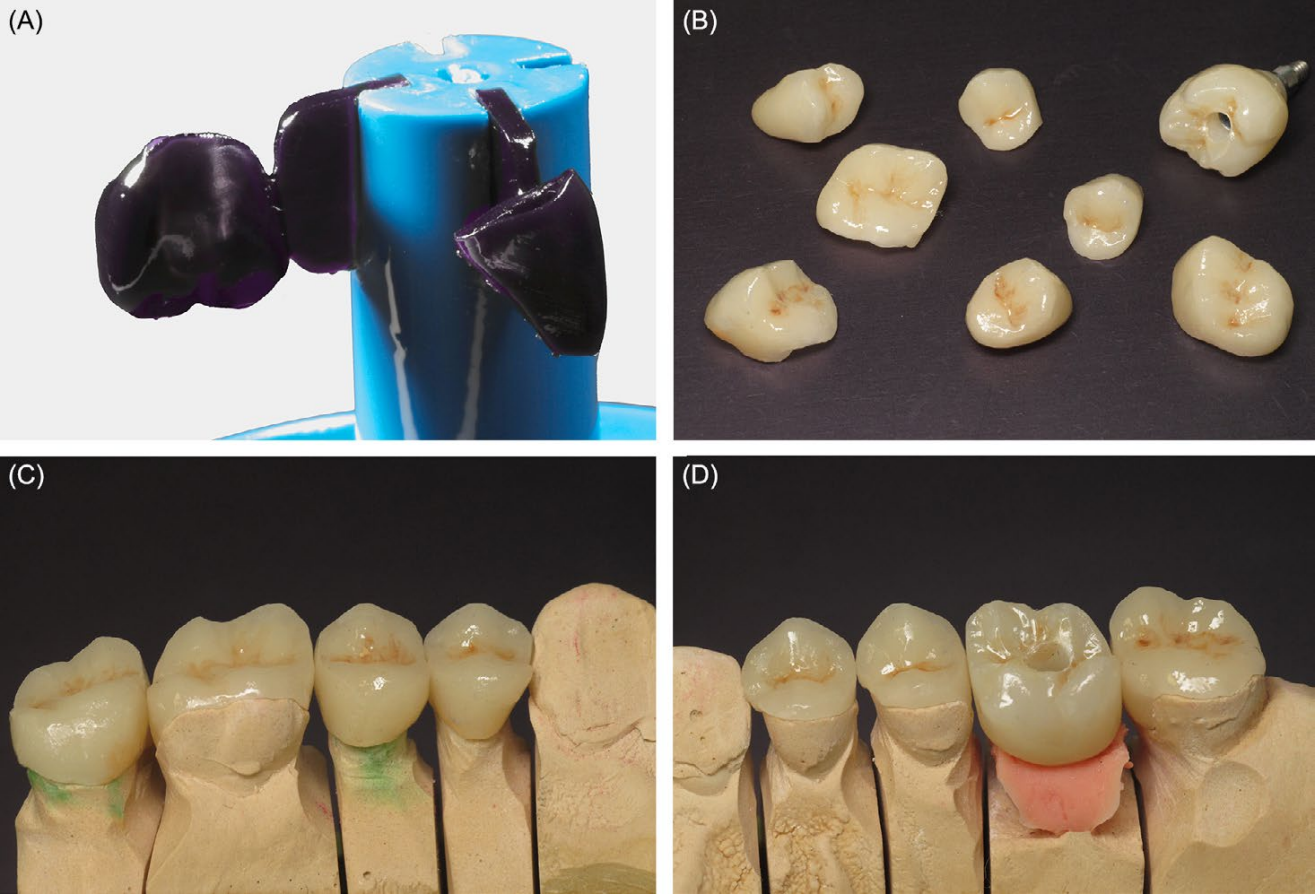
(A) Patterns on sprues before investment with a refractory material. (B) Characterized ceramic restorations. Fit of heat pressed ceramic restorations on dies, on maxillary (C) right and (D) left molars and premolars.

The dental technician performed a final check of the patterns and made any necessary corrections. Final ceramic restorations (IPS e.max Press; Ivoclar Vivadent) were made using the lost‐wax casting process on wax‐resin patterns. A multi‐layer ingot casting technique was used. The refractory material was roughly removed manually and then finely removed by glass bead blasting. Next, the restorations were characterized and finished (Figure 5). The implant‐supported crown was definitively bonded to the abutment.

### 3.6 Positioning of Ceramic Restorations

**FIGURE 6:**
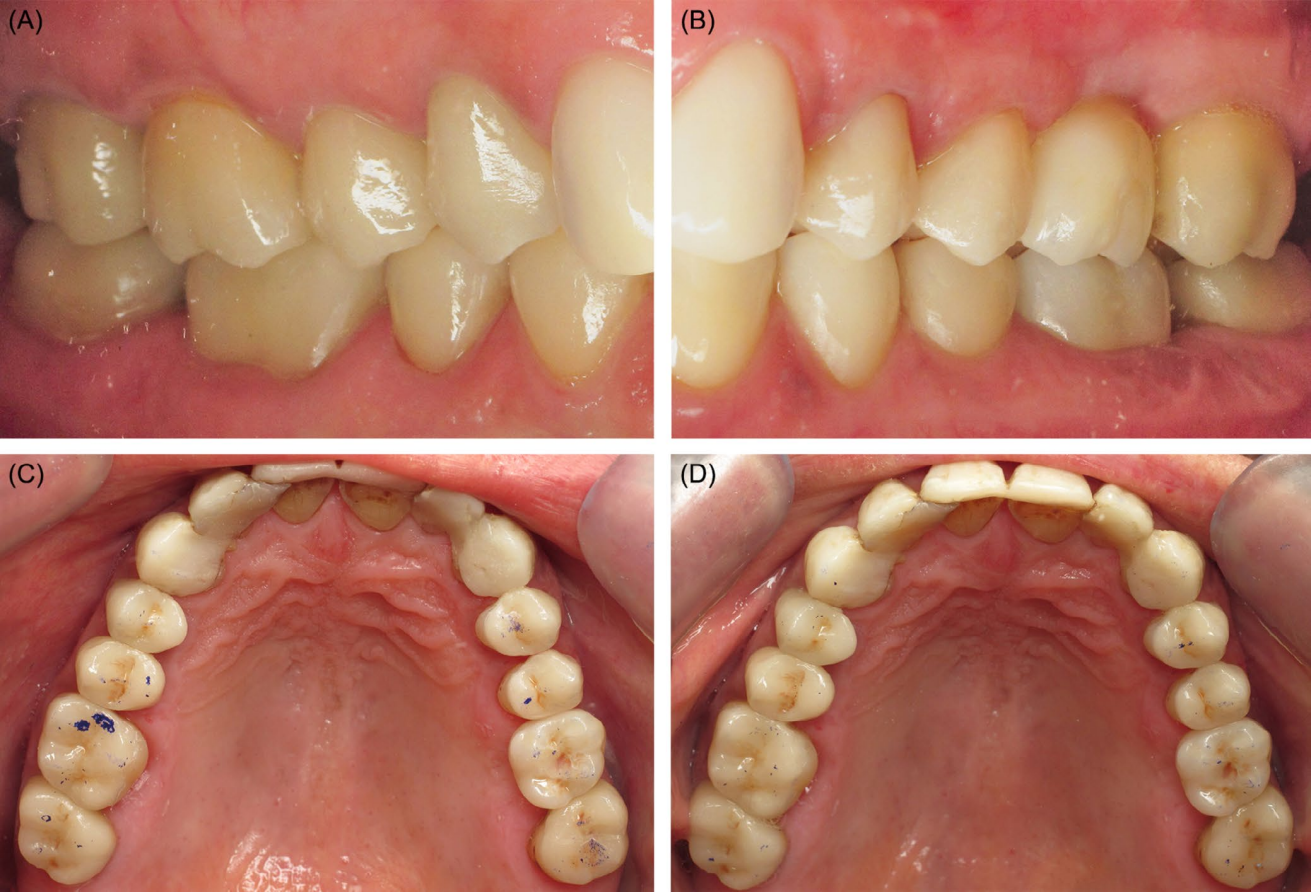
Ceramic restorations fixed on maxillary (A) right and (B) left molars and premolars. Occlusion contacts marked with 12 μm blue articulating paper on fixed restorations (C) before and (D) after occlusal correction. Only right maxillary first molar needed a slight occlusal correction to get back to the original occlusion adjusted on the patterns.

After the interim restorations were removed, the ceramic restorations were tried in the clinical situation. Cervical adjustment and proximal contacts were checked. The restorations were bonded (Optibond XTR and Nexus NX3; Kerr) using a dental dam, and the implant‐supported crown was torqued to the values recommended by the manufacturer (35 Ncm). Then occlusal contacts were verified with 12 μm blue articulating paper and, if necessary, adjusted with a fine diamond grit‐sized burs at slow rotation speed. At this point, only the right maxillary first molar required occlusal adjustment (Figure 6). This adjustment can be attributed to an excess of glaze during hand‐finishing the ceramic restorations. Finally, a meticulous polishing of the adjusted surface was performed.

**FIGURE 7:**
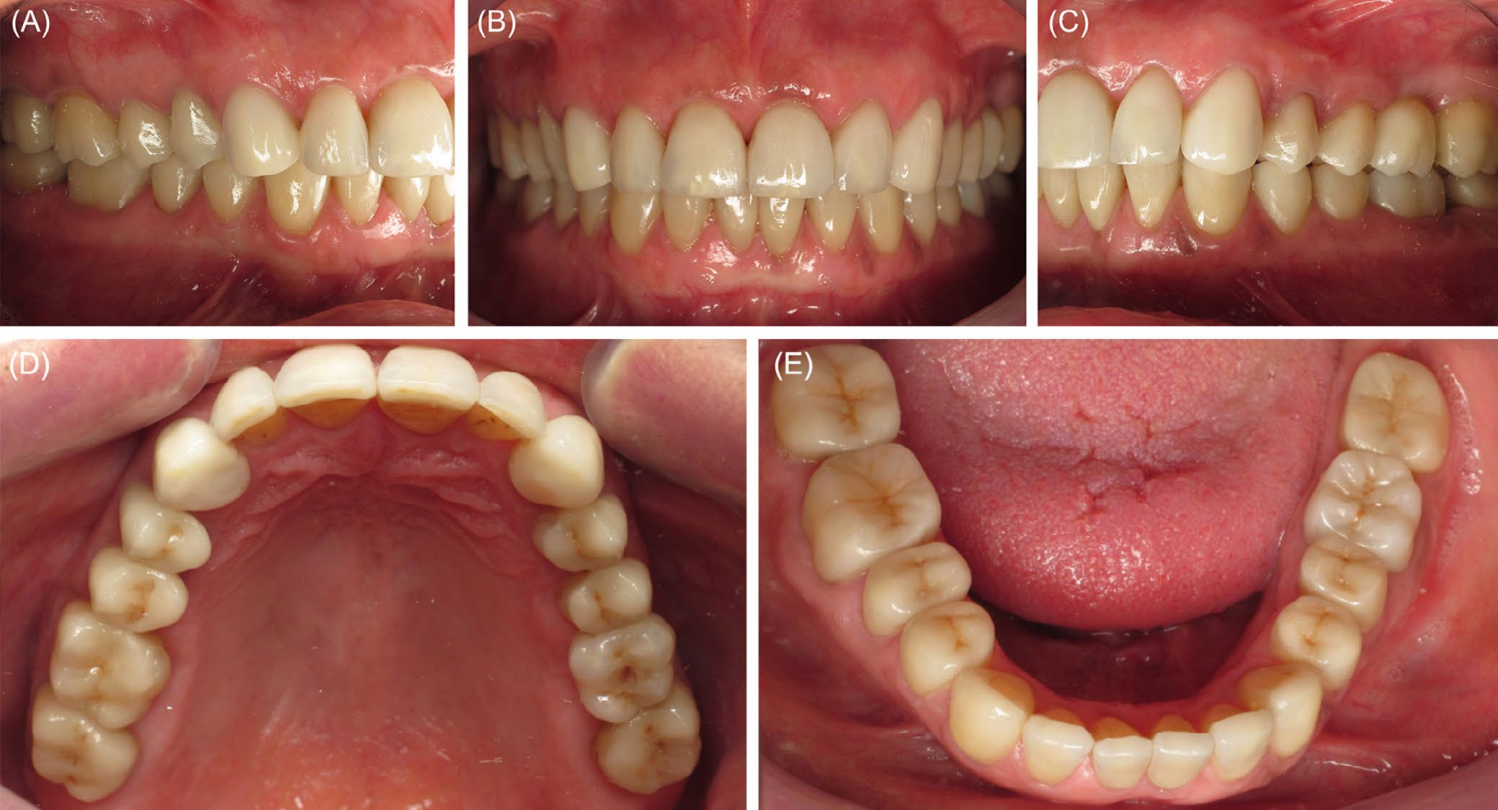
Final intra‐oral situation after complete fixed rehabilitation, (A) right lateral view, (B) front view, (C) left lateral view, (D) maxillary occlusal view, and (E) mandibular occlusal view.

Once this stage was completed, the anterior rehabilitation was carried out according to the initial treatment planification (Figure 7). At 4 years follow‐up, the patient had no complaints and was fully satisfied with the complete mouth rehabilitation.

## 4 Discussion

The main aim of this technique is to minimize final ceramic adjustments. Occlusal adjustments can be critical in extensive fixed prosthetic rehabilitation, particularly with thin or non‐retentive restorations such as overlays, which cannot be tested under occlusal forces prior to bonding due to the risk of fracture [23]. The try‐in of patterns may be an additional step, but it saves considerable time for both the practitioner and dental technician. Resins are easier to adjust than ceramics, and polishing modified ceramics is time‐consuming [24] and cannot restore their mechanical properties [25, 26] or smooth surface [27]. Moreover, grinding weakens ceramic strength [17], and surface roughness reduces flexural strength [28, 29]. Limiting adjustments preserves the mechanical properties and durability of restorations [17]. Pattern try‐in saves time by preventing defects that would require remanufacturing of ceramic restorations.

The wax‐up design is guided by the OVD required to restore the occlusal anatomy, ensuring proper anteroposterior and mediolateral curves. An all‐digital workflow simplifies the integration of these elements, making it easier to replicate the initial design on final restorations [30, 31]. However, challenges may arise during the matching of digital impressions, and limits in superimposition procedures may occur [32]. This workflow also preserves the MMR throughout treatment, reducing errors common in conventional cross‐mounting [30].

Patterns are used as fit and contact checkers and may be easily adjusted by milling or adding castable resin. Milling is done using fine‐grain diamond burs or abrasive discs. Various materials, such as acrylic resins or flowable composites, can modify the shape or cervical limits [33]. The use of the scan‐copy‐mill technique does not affect the choice of apposition material [34], though castable resin allows direct heat‐pressing of ceramics via lost‐wax casting. Any modifications of patterns should be followed by a careful polishing.

However, the adjustability of patterns still has limitations and does not eliminate the need for checking the fit of ceramic restorations. Adjustments may still be required. In addition, patterns must be handled carefully to avoid deformation. Restorations should be inspected by the dental technician before being permanently processed. Depending on the brand of the material, mechanical characteristics can vary significantly. A high modulus of elasticity reduces the risk of pattern deformation during the oral try‐in. The modulus of elasticity of the material used for the presented patterns (Castable Wax Resin; Formlabs) is 220 MPa, while most dental resins exceed 2000 MPa. This difference can be attributed to the inclusion of 20% wax in the material. Therefore, overloading patterns may lead to deformations that could affect functional checks, and the use of pure waxes should be avoided due to the risk of deformation during the in‐mouth try‐in [13]. Thus, the occlusal check should be performed under a gentle bite, and patterns should not be cast in case of excessive defects. Large adjustments should be avoided as they may stem from prior inaccuracies. Correcting the cause of defects and confecting new patterns is more suitable. Patterns may also suffer from insufficient retention, particularly in the case of non‐retentive restorations. In such cases, a fit checker or denture adhesive can enhance stability and retention during the try‐in [13].

The materials are available in a wide range of colors, including VITA shades, transparent, or bright colors. The choice of color is not of great impact in the exposed technique, but it should be noted that the transparent color disrupts the legibility of shapes and occlusal contacts. In addition, VITA shades help visualize restorations but are not useful for color try‐in, as the final ceramic color may differ. White articulating paper may be recommended, as red or blue papers can be difficult to visualize on dark patterns.

The use of patterns is particularly useful for heat‐pressed ceramics using the lost‐wax technique, but restorations can also be milled after scanning patterns [13, 35]. Milled or printed patterns can achieve a fit comparable to conventional methods [36, 37, 38, 39, 40, 41, 42]. The variety of materials and processes complicates workflow comparisons [42], and the accuracy of final restorations depends on many factors, including process rigor and dental technician experience. In addition, very few articles on the use of the patterns as clinical try‐ins have been published to date [12, 13].

Using patterns for clinical try‐in is not a substitute for final restoration checks. This technique is most relevant in extensive rehabilitation, where multiple restorations require significant adjustments, or when the thickness of restorations does not allow try‐in before bonding. In less complex cases, this additional step may provide only limited advantages. Further studies are needed to quantify the benefits of this procedure.

## 5 Conclusion

This protocol is intended to reduce ceramic adjustments on definitive restorations. The necessary adjustments are made directly on the patterns to prevent potential significant defects. Depending on the manufacturing processes of restorations, particular attention must be given to the type of material used for the patterns. Printable castable wax and resin patterns are particularly interesting for the manufacture of heat‐pressed ceramic restorations. The time consumed during the wax and resin patterns try‐in is overall beneficial for both the practitioner and the dental technician. Clinical try‐in of patterns is indicated for global prosthetic rehabilitations, as the large number of restorations increases the risk of defects and requires numerous esthetic and functional controls.

## Author Contributions

**Etienne Lefrançois:** conceptualization, investigation, methodology, visualization, writing – original draft, writing – review and editing. **Ludovic Aubault:** conceptualization, investigation, methodology, resources. **Salomé Provost:** methodology, visualization, writing – original draft, writing – review and editing.

## Consent

Written informed consent was obtained from the patient to publish this report in accordance with the journal's patient consent policy.

## Conflicts of Interest

The authors declare no conflicts of interest.

## Data Availability

The data that support the findings of this study are available from the corresponding author upon reasonable request.

## References

[1] P. Boitelle , B. Mawussi , L. Tapie , and O. Fromentin , “A Systematic Review of CAD/CAM Fit Restoration Evaluations,” Journal of Oral Rehabilitation 41, no. 11 (2014): 853–874, 10.1111/joor.12205. 24952991

[2] M. Hasanzade , M. Aminikhah , K. I. Afrashtehfar , and M. Alikhasi , “Marginal and Internal Adaptation of Single Crowns and Fixed Dental Prostheses by Using Digital and Conventional Workflows: A Systematic Review and Meta‐Analysis,” Journal of Prosthetic Dentistry 126, no. 3 (2021): 360–368, 10.1016/j.prosdent.2020.07.007. 32928518

[3] I. B. Sanches , T. C. Metzker , R. Kappler , M. V. Oliveira , A. O. Carvalho , and E. M. Castor Xisto Lima , “Marginal Adaptation of CAD‐CAM and Heat‐Pressed Lithium Disilicate Crowns: A Systematic Review and Meta‐Analysis,” Journal of Prosthetic Dentistry 129, no. 1 (2023): 34–39, 10.1016/j.prosdent.2021.03.021. 34147239

[4] S. Papadiochou and A. L. Pissiotis , “Marginal Adaptation and CAD‐CAM Technology: A Systematic Review of Restorative Material and Fabrication Techniques,” Journal of Prosthetic Dentistry 119, no. 4 (2018): 545–551, 10.1016/j.prosdent.2017.07.001. 28967399

[5] T. Joda , S. Wolfart , S. Reich , and N. Zitzmann , “Virtual Dental Patient: How Long Until It's Here?,” Current Oral Health Reports 5 (2018): 1–5, 10.1007/s40496-018-0178-y.

[6] M. Revilla‐León , R. Agustín‐Panadero , J. M. Zeitler , et al., “Differences in Maxillomandibular Relationship Recorded at Centric Relation When Using a Conventional Method, Four Intraoral Scanners, and a Jaw Tracking System: A Clinical Study,” Journal of Prosthetic Dentistry 132, no. 5 (2024): 964–972, 10.1016/j.prosdent.2022.12.007. 36682896

[7] C. Raffone , F. Gianfreda , M. G. Pompeo , D. Antonacci , P. Bollero , and L. Canullo , “Chairside Virtual Patient Protocol. Part 2: Management of Multiple Face Scans and Alignment Predictability,” Journal of Dentistry 122 (2022): 104123, 10.1016/j.jdent.2022.104123. 35413410

[8] P. Magne and U. C. Belser , “Novel Porcelain Laminate Preparation Approach Driven by a Diagnostic Mock‐Up,” Journal of Esthetic and Restorative Dentistry 16, no. 1 (2004): 7–16, 10.1111/j.1708-8240.2004.tb00444.x. 15259539

[9] G. Gurel , S. Morimoto , M. A. Calamita , C. Coachman , and N. Sesma , “Clinical Performance of Porcelain Laminate Veneers: Outcomes of the Aesthetic Pre‐Evaluative Temporary (APT) Technique,” International Journal of Periodontics and Restorative Dentistry 32, no. 6 (2012): 625–635. 23057051

[10] G. Fabbri , G. Cannistraro , C. Pulcini , and R. Sorrentino , “The Full‐Mouth Mock‐Up: A Dynamic Diagnostic Approach (DDA) to Test Function and Esthetics in Complex Rehabilitations With Increased Vertical Dimension of Occlusion,” International Journal of Esthetic Dentistry 13, no. 4 (2018): 460–474. 30302437

[11] G. E. Goll , “Production of Accurately Fitting Full‐Arch Implant Frameworks: Part I—Clinical Procedures,” Journal of Prosthetic Dentistry 66, no. 3 (1991): 377–384, 10.1016/0022-3913(91)90266-y. 1800736

[12] C. Jurado and D. Givan , “Printed Wax‐Up for Intra‐Oral Try‐In,” Poster presented at: 91st Annual Scientific Meeting of the American Prosthodontic Society, Chicago, USA, February 22, 2019, 10.13140/RG.2.2.24763.64806.

[13] C. Brenes , C. S. Babb , S. Bencharit , M. Romeroc , and R. Arced , “Digital Approach to the Fabrication of a Wax Prototype for Full‐Mouth Rehabilitation of a Worn Dentition: A Clinical Report,” Journal of Oral Science Rehabilitation 3, no. 4 (2017): 44–49.

[14] F. Cattoni , G. Teté , A. M. Calloni , F. Manazza , G. Gastaldi , and P. Capparè , “Milled Versus Moulded Mock‐Ups Based on the Superimposition of 3D Meshes From Digital Oral Impressions: A Comparative In Vitro Study in the Aesthetic Area,” BMC Oral Health 19, no. 1 (2019): 230, 10.1186/s12903-019-0922-2. 31664999 PMC6819647

[15] A. Lo Giudice , L. Ortensi , M. Farronato , A. Lucchese , E. Lo Castro , and G. Isola , “The Step Further Smile Virtual Planning: Milled Versus Prototyped Mock‐Ups for the Evaluation of the Designed Smile Characteristics,” BMC Oral Health 20, no. 1 (2020): 165, 10.1186/s12903-020-01145-z. 32503567 PMC7275593

[16] L. Ortensi , E. Lo Castro , E. Rapisarda , and E. Pedullà , “Accuracy of Trial Restorations From Virtual Planning: A Comparison of Two Fabrication Techniques,” Journal of Prosthetic Dentistry 127, no. 3 (2022): 425–429, 10.1016/j.prosdent.2020.08.040. 33317829

[17] A. Coldea , J. Fischer , M. V. Swain , and N. Thiel , “Damage Tolerance of Indirect Restorative Materials (Including PICN) After Simulated Bur Adjustments,” Dental Materials 31, no. 6 (2015): 684–694, 10.1016/j.dental.2015.03.007. 25858782

[18] R. Ahmad , S. M. Morgano , B. M. Wu , and R. A. Giordano , “An Evaluation of the Effects of Handpiece Speed, Abrasive Characteristics, and Polishing Load on the Flexural Strength of Polished Ceramics,” Journal of Prosthetic Dentistry 94, no. 5 (2005): 421–429, 10.1016/j.prosdent.2005.08.014. 16275301

[19] C. W. Chang , J. N. Waddell , K. M. Lyons , and M. V. Swain , “Cracking of Porcelain Surfaces Arising From Abrasive Grinding With a Dental Air Turbine,” Journal of Prosthodontics 20, no. 8 (2011): 613–620, 10.1111/j.1532-849X.2011.00760.x. 22017480

[20] S. Rues , F. S. Schwindling , A. Meyer , P. Rammelsberg , and M. Schmitter , “Fracture Resistance of Zirconia‐Based All‐Ceramic Crowns After Bur Adjustment,” European Journal of Oral Sciences 125, no. 4 (2017): 310–313, 10.1111/eos.12353. 28597965

[21] X. F. Song and L. Yin , “The Quantitative Effect of Diamond Grit Size on the Subsurface Damage Induced in Dental Adjustment of Porcelain Surfaces,” Proceedings of the Institution of Mechanical Engineers. Part H 224, no. 10 (2010): 1185–1194, 10.1243/09544119JEIM737. 21138236

[22] X. F. Song and L. Yin , “Stress and Damage at the Bur‐Prosthesis Interface in Dental Adjustments of a Leucite‐Reinforced Glass Ceramic,” Journal of Oral Rehabilitation 37, no. 9 (2010): 680–691, 10.1111/j.1365-2842.2010.02099.x. 20492439

[23] E. B. S. Valenzuela , J. P. Andrade , P. F. J. S. da Cunha , H. R. Bittencourt , and A. M. Spohr , “Fracture Load of CAD/CAM Ultrathin Occlusal Veneers Luted to Enamel or Dentin,” Journal of Esthetic and Restorative Dentistry 33, no. 3 (2021): 516–521, 10.1111/jerd.12658. 32949221

[24] T. M. da Silva , A. C. R. D. Salvia , R. F. de Carvalho , C. Pagani , D. M. da Rocha , and E. G. da Silva , “Polishing for Glass Ceramics: Which Protocol?,” Journal of Prosthodontic Research 58, no. 3 (2014): 160–170, 10.1016/j.jpor.2014.02.001. 24684959

[25] M. Guazzato , M. Albakry , L. Quach , and M. V. Swain , “Influence of Grinding, Sandblasting, Polishing and Heat Treatment on the Flexural Strength of a Glass‐Infiltrated Alumina‐Reinforced Dental Ceramic,” Biomaterials 25, no. 11 (2004): 2153–2160, 10.1016/j.biomaterials.2003.08.056. 14741630

[26] M. Schmitter , G. Lotze , W. Bömicke , and S. Rues , “Influence of Surface Treatment on the In‐Vitro Fracture Resistance of Zirconia‐Based All‐Ceramic Anterior Crowns,” Dental Materials 31, no. 12 (2015): 1552–1560, 10.1016/j.dental.2015.10.003. 26547870

[27] S. P. Amaya‐Pajares , A. V. Ritter , C. Vera Resendiz , B. R. Henson , L. Culp , and T. E. Donovan , “Effect of Finishing and Polishing on the Surface Roughness of Four Ceramic Materials After Occlusal Adjustment,” Journal of Esthetic and Restorative Dentistry 28, no. 6 (2016): 382–396, 10.1111/jerd.12222. 27264939

[28] N. de Jager , A. J. Feilzer , and C. L. Davidson , “The Influence of Surface Roughness on Porcelain Strength,” Dental Materials 16, no. 6 (2000): 381–388, 10.1016/s0109-5641(00)00030-0. 10967186

[29] H. Fischer , M. Schäfer , and R. Marx , “Effect of Surface Roughness on Flexural Strength of Veneer Ceramics,” Journal of Dental Research 82, no. 12 (2003): 972–975, 10.1177/154405910308201207. 14630897

[30] M. Valenti and J. H. Schmitz , “A Reverse Digital Workflow by Using an Interim Restoration Scan and Patient‐Specific Motion With an Intraoral Scanner,” Journal of Prosthetic Dentistry 126, no. 1 (2021): 19–23, 10.1016/j.prosdent.2020.05.011. 32763090

[31] N. Sinada and C. I. Wang , “Fixed Prosthodontic Rehabilitation With a Fully Digital Workflow for a Teenage Patient With Amelogenesis Imperfecta: A 2‐Year Follow‐Up,” Journal of Prosthetic Dentistry 131, no. 1 (2024): 1–6, 10.1016/j.prosdent.2022.02.025. 35473905

[32] W. Cascón , V. Hsu , and M. Revilla‐León , “Facially Driven Digital Diagnostic Waxing: New Software Features to Simulate and Define Restorative Outcomes,” Current Oral Health Reports 6, no. 4 (2019): 284–294, 10.1007/s40496-019-00233-6.

[33] D. R. Burns , D. A. Beck , and S. K. Nelson , “A Review of Selected Dental Literature on Contemporary Provisional Fixed Prosthodontic Treatment: Report of the Committee on Research in Fixed Prosthodontics of the Academy of Fixed Prosthodontics,” Journal of Prosthetic Dentistry 90, no. 5 (2003): 474–497, 10.1016/S0022-3913(03)00259-2. 14586312

[34] K. Tokumoto , T. Mino , Y. Kurosaki , et al., “Fixed Partial Denture Designed by Combining the Whole 3D Digital Surface Morphology of the Provisional Restoration and Abutment Teeth Surfaces,” Acta Medica Okayama 76, no. 1 (2022): 79–84, 10.18926/AMO/63215. 35237002

[35] A. Agnini , D. Romeo , B. Giulia , W. Tommaso , C. Christian , and A. Agnini , “Copy–Paste Concept: Full Digital Approach in the Management of Gingival Emergence Profiles,” Journal of Esthetic and Restorative Dentistry 35, no. 1 (2023): 222–229, 10.1111/jerd.13014. 36633264

[36] H. M. Fathi , A. H. Al‐Masoody , N. El‐Ghezawi , and A. Johnson , “The Accuracy of Fit of Crowns Made From Wax Patterns Produced Conventionally (Hand Formed) and via CAD/CAM Technology,” European Journal of Prosthodontics and Restorative Dentistry 24, no. 1 (2016): 10–17. 27039473

[37] A. Sepulveda , F. Guerrero‐Martínez , C. Gaitan‐Fonseca , T. Komabayashi , E. Reyes‐Vela , and D. Masuoka , “Evaluation of the Marginal Adaptation in Metal Crowns Using CAD/CAM and Manual Wax Patterns,” Microscopy Research 3, no. 2 (2015): 26–32, 10.4236/mr.2015.32004.

[38] F. R. Homsy , M. Özcan , M. Khoury , and Z. A. K. Majzoub , “Marginal and Internal Fit of Pressed Lithium Disilicate Inlays Fabricated With Milling, 3D Printing, and Conventional Technologies,” Journal of Prosthetic Dentistry 119, no. 5 (2018): 783–790, 10.1016/j.prosdent.2017.07.025. 28969918

[39] L. Shamseddine , R. Mortada , K. Rifai , and J. J. Chidiac , “Marginal and Internal Fit of Pressed Ceramic Crowns Made From Conventional and Computer‐Aided Design and Computer‐Aided Manufacturing Wax Patterns: An In Vitro Comparison,” Journal of Prosthetic Dentistry 116, no. 2 (2016): 242–248, 10.1016/j.prosdent.2015.12.005. 26948080

[40] L. Shamseddine , R. Mortada , K. Rifai , and J. J. Chidiac , “Fit of Pressed Crowns Fabricated From Two CAD‐CAM Wax Pattern Process Plans: A Comparative In Vitro Study,” Journal of Prosthetic Dentistry 118, no. 1 (2017): 49–54, 10.1016/j.prosdent.2016.10.003. 28024815

[41] F. R. Homsy , M. Özcan , M. Khoury , and Z. A. K. Majzoub , “Comparison of Fit Accuracy of Pressed Lithium Disilicate Inlays Fabricated From Wax or Resin Patterns With Conventional and CAD‐CAM Technologies,” Journal of Prosthetic Dentistry 120, no. 4 (2018): 530–536, 10.1016/j.prosdent.2018.04.006. 30318049

[42] L. Guachetá , C. D. Stevens , J. A. Tamayo Cardona , and R. Murgueitio , “Comparison of Marginal and Internal Fit of Pressed Lithium Disilicate Veneers Fabricated via a Manual Waxing Technique Versus a 3D Printed Technique,” Journal of Esthetic and Restorative Dentistry 34, no. 4 (2022): 715–720, 10.1111/jerd.12675. 33174306

